# Promoting experiential learning through the use of high-fidelity human patient simulators in midwifery: A qualitative study

**DOI:** 10.4102/curationis.v42i1.1882

**Published:** 2019-01-21

**Authors:** Hafaza B. Amod, Petra Brysiewicz

**Affiliations:** 1 Discipline of Nursing, School of Nursing and Public Health, University of KwaZulu-Natal, South Africa

## Abstract

**Background:**

The need to use innovative teaching and learning strategies in the nursing pedagogy is important in the 21st century. The challenges of clinical sites and opportunities for nursing students to gain clinical experience are a growing concern for many nurse educators. High-fidelity human patient simulators (HFHPS) are computerised mannequins that replicate a real-life patient, and when integrated into classroom teaching they allow students to become fully immersed into an almost real-life scenario.

**Objectives:**

The aim of this study was to describe how HFHPS can promote experiential learning following the management of postpartum haemorrhage as a midwifery clinical emergency.

**Method:**

A descriptive qualitative research approach was carried out in this study. The research setting was a local university in KwaZulu-Natal. The total population included all (*N* = 43) fourth-year baccalaureate of nursing undergraduate student midwives who participated as observers and/or role-players of a scenario role-play. An all-inclusive sampling was performed. There were 43 student midwives involved in the simulation teaching session with 6 of these students actively participating in each role-play at a time, while the remaining 37 observed. This occurred in two separate sessions and all the student midwives were involved in a debriefing session. These student midwives were then followed up and asked to participate in a focus group. The data in this article came from two separate focus groups which comprised 20 student midwives in total. Data were analysed using content analysis.

**Results:**

Four categories emerged from the data, namely HFHPS offers a unique opportunity for student midwives to manage complex real-life emergencies; promotes reflection by allowing student midwives to reflect or review their roles, decisions and skills; allows student midwives to learn from their own experiences and encourages student midwives to try out what they learnt in a real-life situation.

**Conclusion:**

High-fidelity human patient simulators can be used in a complex case scenario to promote experiential learning of a clinical emergency.

## Introduction

The development of fundamental clinical skills is an important component in preparing students to meet the responsibilities of a midwife. Findings from studies conducted by Linder and Pulsipher (2008) and Sundler, Petterson and Berglund (2015) revealed that simulation has become an established pedagogy for teaching clinical skills, thus providing students the opportunity to gain essential skills in a ‘real-life’-like environment. The need to use innovative teaching and learning strategies in the nursing pedagogy is important in the 21st century. The challenges of clinical sites and opportunities for nursing students to gain clinical experience are a growing concern for many nurse educators. Simulation experiences using high-fidelity human patient simulators (HFHPS) provide an effective teaching and learning approach as they allow students to become active and fully participative learners (Jeffries & Jeffries 2012; Powell-Laney, Keen & Hall 2012). High-fidelity human patient simulators are computerised mannequins that imitate real-life signs and symptoms such as breathing, pulse, blood pressure, moaning and blinking to enhance realism (Flood et al. 2011; Jeffries & Jeffries 2012). This type of technology allows nurse educators an opportunity to design various case scenarios using advanced equipment and without fear of compromising patient safety. Through active learning or engagement, students are offered an opportunity to practise rare and/or complex emergencies in a safe and controlled environment (Boet et al. 2014; Lewis, Strachan & Smith 2012).

Jeffries and Jeffries (2012) state that ensuring active engagement in the learning process is important when designing and using simulation. Students can practise various procedural tasks and complex cognitive skills in a simulated setting and these skills can transfer to the workplace, thus enhancing patient care (Bruppacher et al. 2010; Phipps et al. 2012; Riley et al. 2011).

Getting students to ‘learn by doing’ offers students the opportunity to understand easy and complex skills through active participation (Farooq 2013). Kolb (2014) viewed learning as a continuous process of knowledge creation through the transformation of experience. Kolb’s experiential learning theory (2014) addresses the provision of learning experience to meet the needs of all types of learners. He describes how information processing and learning occurs in cycles, and this process starts with concrete experiences in which students immerse themselves fully into a new experience that they consider to be stimulating or challenging. The second phase of reflective observation occurs when students observe and reflect on their concrete experience. This follows with the creation of logical theories through the integration of observation, which is termed as an abstract conceptualisation. The last phase, which is active experimentation, is when students apply these new theories during decision-making and problem-solving activities. When learners are able to execute all four stages of the cycle, effective learning takes place (Chan 2012; Stocker, Burmester & Allen 2014; Wolfgram & Quinn 2013). According to Zigmont, Kappus and Sudikoff (2011), the experiential learning process involves participation and analysis of key experiences. Yardley, Teunissen and Dornan (2012) define experiential learning as the construction of knowledge and meaning from real-life experiences. Hence, experiential learning is situated in a context that is relevant to the learners’ own future careers.

In this study, the researcher used real-life people to play the roles of various healthcare team members as practised in a real-life emergency. Student midwives played the role of a registered midwife, enrolled nurse, student midwife, a medical doctor and a birth companion (Table 1). The scenario offered student midwives an opportunity to understand their independent roles as well as the importance of teamwork during the management of an obstetric emergency.

**TABLE 1:** Gender demographics of participants.

Variables	Scenario 1	Scenario 2
**Role-players**		
Team leader (registered midwife)	Female	Female
Assistant 1 (student midwife)	Male	Female
Assistant 2 (student midwife)	Male	Female
Enrolled nurse	Female	Female
Husband (birth companion)	Male	Male
Doctor	Male	Male
**Observers**		
37 students	3 Male	5 Male
	34 Female	32 Female
**Debriefing**		
All participants (*N* = 43)	36 Female	7 Male
Focus group 1	6 Female	6 Female
Focus group 2	6 Male	2 Male

### Problem statement

Various midwifery clinical skills are complex in nature and difficult to teach during an actual emergency. Postpartum haemorrhage (PPH), which is the most common type of obstetric emergency, accounts for 25% of maternal deaths worldwide and the third most common cause of maternal mortality in South Africa (National Committee for Confidential Enquiries into Maternal Deaths 2014). Simulation of PPH as an emergency could be used to improve patient care outcomes by offering students an opportunity to improve their performance while reflecting on and learning from their mistakes until they get it right and before managing actual patients with the same emergency (Lestander, Lehto & Engstrom 2016; Ness et al. 2010). The scenario on PPH requires prompt application of good clinical, decision-making and problem-solving skills. Students are required to think fast and use a combination of skills to manage their simulated patient successfully. According to Jeffries and Jeffries (2012), this type of complex learning promotes high expectations for both the learners and the educators.

Using the four phases of the experiential learning cycle, as described by Kolb (2014), this study aims to explore and describe the experiences of student midwives who used HFHPS in a PPH case scenario. The experiential learning cycle was used as a conceptual framework for data analysis, and therefore the findings of this study are linked to each phase of the cycle.

### Objective of the study

The aim of the study was to explore how HFHPS can promote experiential learning of a midwifery clinical emergency such as PPH.

### Definition of terms

High-fidelity human patient simulators are computer-controlled mannequins that are designed to mimic an almost real-life interaction with students in a controlled simulated clinical setting (Jeffries & Jeffries 2012).

Experiential learning is the construction of knowledge and meaning from real-life experiences related to a learner’s future career (Yardley et al. 2012).

### Significance of the study

The importance of this study is to explore experiential learning during classroom teaching by using HFHPS to enable students to manage an obstetric emergency such as PPH in the clinical setting.

## Research approach

The researcher used a descriptive qualitative research approach to explore the views of student midwives who used HFHPS to learn to manage an obstetric emergency in a clinical skills laboratory.

### Research setting

The research setting was a local university in KwaZulu-Natal, South Africa. The university offers a variety of nursing speciality programmes both at undergraduate and postgraduate (master’s) level. The basic midwifery module is offered in the fourth year of the undergraduate programme. The nursing discipline has a fully functional clinical skills laboratory with high-, medium- and low-fidelity human patient simulators used in the teaching of nursing and medical students.

### Research participants

The research participants were all fourth-year undergraduate student midwives (total population = 43) who were enrolled in the midwifery module within an undergraduate baccalaureate of nursing programme. An all-inclusive sampling method was used in this study. Two groups of 6 student midwives (12 in total) participated in different roles in the PPH scenario (Table 1). The different roles in the team were deliberately included to maintain the realness of the situation and highlight the importance of teamwork when managing emergencies. The remaining 37 student midwives observed the scenario.

### Data collection process

In 2015, the researcher and the research supervisor, after an extensive literature search, developed a draft simulation learning package on PPH. The draft package was evaluated by a team of experts consisting of midwifery clinical specialists, midwifery educators, nursing education specialists and obstetricians within South Africa. The package was subsequently improved and used to teach undergraduate student midwives who were in the 2016 group. These student midwives were expected to read the guidelines and instructions in the package and volunteer to participate in a PPH scenario role-play. Two groups of six undergraduate student midwives took part in the PPH scenario role-play which used a HFHPS (SimMom). The simulation experience comprised a briefing session prior to commencing, the scenario on PPH and then a debriefing session. Although the majority of the participants were eager to participate, only six role-players per session were required for the scenario. The roles included that of a registered midwife (who was the team leader), two student midwives who were assistants to the team leader, a staff nurse to assist in recordkeeping, a husband who offered moral support to his wife (SimMom) and a doctor to show collaboration with medical staff during obstetric emergencies. These role-players were selected to authenticate a real-life setup of a PPH scenario in a labour or delivery suite. The researcher, together with the remaining 37 student midwives observed the simulation session in real time, via a video conferencing system. Each scenario lasted approximately 13 minutes. The debriefing session in the classroom occurred immediately after the scenario exercise. The purpose of the debriefing was to explore the experiences of student midwives who either role-played or observed the scenario on PPH. This included reflecting on the actions and/or decisions made by themselves or by their peers by identifying gaps related to the actual management of PPH (Amod & Brysiewicz 2017).

Following the educational activity, the researcher conducted and audio-recorded two separate focus group discussions to explore the views of student midwives regarding the PPH simulation learning experience. The first focus group (FG1) was conducted on the same day as the simulation exercise and included all the 12 participants who were role-players in the scenario. The researcher asked open-ended questions, which were linked to experiential learning (Box 1). The second focus group (FG2) was conducted a week later in the clinical area, and included another eight student midwives.

BOX 1 Questions posed in focus group.

1. How would you describe your experience in the simulation scenario?
2. Do you feel that the simulation scenario was beneficial to you?
  2.1. If yes, what are some of the benefits that you identified?
  2.2. If no, how can we improve the simulation scenario?
3. How do you feel about being able to manage a patient with postpartum haemorrhage following exposure to this type of learning?
4. Did the simulation scenario improve any skills?
  4.1. If yes, what skills were challenged in the scenario?
  4.2. How did the participation in the simulation affect your self-confidence?
5. Would you like to be involved in other midwifery simulation learning?

### Data analysis

The data for this article were extracted from two separate focus groups which comprised 20 student midwives who participated as observers and role-players of the PPH scenario. The recordings of the focus groups were transcribed. Transcriptions were analysed by the researcher and the research supervisor using content analysis as described by Graneheim, Lindgren and Lundman (2017).

### Trustworthiness

According to Cope (2014), trustworthiness refers to the quality value of the results and conclusions reached in a qualitative study. In this study, the researcher knew the participants who were fourth-year student midwives enrolled in the baccalaureate programme. A good rapport developed between the participants and the researcher who was their teacher and clinical facilitator. Participants were relaxed and comfortable throughout the data collection process.

The simulated scenario setup on PPH occurred under an almost natural circumstance, so that student midwives would interact in an almost real-life clinical situation. This was to provide sufficient details and to give the reader a sense of the path taken by the researcher. The researcher posed her pre-planned questions, during the debriefing and focus group sessions. Participants were encouraged to express their views on their experience freely. The researcher provides an -in-depth description to give the reader a sense of ‘being there’, as well as attempts to demonstrate that the knowledge acquired may be relevant to a similar situation. All data were analysed with the guidance of the research supervisor with whom frequent sessions were held to interpret the results.

### Ethical considerations

The University Research Committee granted permission to conduct this study (reference number HSS/0320/015M). Each participant was provided an information sheet, explaining the purpose and nature of the study, prior to obtaining written consent. The participants were informed of their right to withdraw voluntarily at any stage of the study and that non-participation would not affect their education or classes in any manner. The right to privacy and confidentiality was maintained throughout the study.

## Results

### Demographic data

Forty-three undergraduate student midwives participated in the educational activity. There were 7 male and 36 female participants ranging between 21 and 35 years of age. Table 1 shows the gender demographics of participants of the educational activity. Of the total population, there were 12 student midwives who participated as role-players in a scenario on PPH. These 12 students were included in the FG1. The remaining student midwives observed the scenario together with the researcher. A FG2 was conducted a week later and included eight student midwives who participated as either role-players or observers of the PPH scenario. A total of 20 student midwives were included in the focus groups.

The data from these focus groups were coded and categorised using content analysis. The four categories that emerged from findings are discussed.

#### Phase 1: Managing real-life emergencies (the concrete experience)

The scenario on PPH incorporated the use of a high-fidelity mannequin (SimMom) which allowed student midwives an opportunity to experience an almost real-life emergency in a confined environment. Each role-player received an action card, which explained their role and expectations in the simulation exercise.

**Theme 1: Good simulation of real complex environment:** The scenario on PPH is a complex case scenario, which requires a combination of skills such as clinical competence, decision-making and problem-solving skills as is needed in real-life situations. These competencies are essential to learn to manage obstetric emergencies successfully. The scenario offered a unique opportunity for situated learning by allowing student midwives to practise realistic scenarios while becoming fully immersed in the situation. One participant role-player said:

‘It [*the scenario*] was exciting and turned out good…I liked how decisions were made whilst waiting for the doctor.’ (FG1, P1, female)

Another participant role-player added:

‘It [*the scenario*] was good. Students were relaxed. They explained each step and it was nice to watch. I did not see a PPH case in real life, but this simulation helped me form an imagination on how I will do it in real life.’ (FG1, P6, female)

The distinct roles clearly outlined inter-professional teamwork and added to the realness of the situation. These team roles are similar to what student midwives experience in their placement hospitals where such emergencies are common. One participant observer commented:

‘It was a good practical experience. SimMom responds likes a real patient. You can speak to the SimMom and…monitor the vital signs.’ (FG2, P6, female)

A second observer added:

‘It is different from doing a competency, like we have been doing in the past years…with simulation you are able to learn whatever theory and then apply the practical.’ (FG2, P4, female)

**Theme 2: Simulation of a real environment may be challenging:** Although most student midwives spoke about how they enjoyed the high-fidelity simulation experience, one student midwife stated that the experience was challenging. The role-player said:

‘I found the experience to be challenging as I played the role of the doctor so I applied my learning experience.’ (FG1, P2, male)

#### Phase 2: Promoting reflection (reflective observation)

Feedback is an essential aspect of simulation as it allows students to reflect on their roles, decisions and skills from their own learning experiences.

**Theme 3: Reflection, though challenging, is helpful in the learning process:** In this study, participants were encouraged to discuss their experience during a debriefing.

One participant observer said:

‘I felt that it was very helpful…getting the students to do the simulation itself is a good way for us to practise our skills and it helped me.’ (FG2, P1, female)

The results showed that participants found the experience to be new and helpful. They were able to assess their own performances and that of their peers.

One participant role-player said:

‘It [*the simulation*] was an eye opener. I was able to identify gaps. It was similar to a real-life situation and very beneficial to my knowledge.’ (FG1, P6, female)

#### Phase 3: Thinking about learning (abstract conceptualisation)

The purpose of using a high-fidelity simulator in a realistic scenario helps student midwives to practise a combination of learning outcomes such as knowledge, skills, satisfaction, confidence and critical thinking. When students are able to achieve these outcomes, they feel encouraged to partake in any similar real-life opportunity.

**Theme 4: Facilitating thinking of future application of skills:** These responses show that student midwives were able to reflect on their performance and thus evaluate their learning.

One participant who was the team leader in the scenario said:

‘I was able to delegate effectively making sure the team knew what to do in terms of remembering. It is an easier method to learn from experiencing what is expected in a clinical situation.’ (FG1, P6, female)

Another participant role-player said:

‘After going through the simulation, I found that I understand much more. It helped me to actually know what I was doing and to highlight other areas where I need to develop further.’ (FG1, P5, male)

A third participant role-player said:

‘I feel confident to do it in the ward. The guidance was clear on what to expect in the wards, it may not be the exact same thing, but you will know the steps to follow.’ (FG1, P2, male)

#### Phase 4: Try out what was learnt (active experimentation)

Learning, practising and mastering certain nursing procedures, and using high-fidelity simulation can encourage student midwives to become confident and ‘hands on’ in the practical situation. High-fidelity simulation prepares student midwives to use multiple skills simultaneously to manage an obstetric emergency (PPH) in an almost real-life scenario.

**Theme 5: Linking simulation to practise**: The participants identified that by applying the principles learnt during a scenario experience into a real-life situation, active experimentation can take place.

One participant who was observing the role-play commented:

‘You know when I think back from the experience [*I had*] soon after we started this course, there was a patient having an eclamptic fit. I went [*away*] and started helping other patients because I didn’t know what to do. But now that has changed especially in PPH because you feel that you won’t stand but get involved and help.’ (FG2, P6, female)

Another participant observer added:

‘We also had a case and it was really easy. I helped with catheterising the patient, putting up IV lines and vital signs monitoring. I found it very similar to the simulation. I was able to relate back to it [*the simulation experience*] and see what they [*the role-players*] were saying and doing.’ (FG2, P3, female)

One participant role-player quickly added:

‘The fact that we were shining (giggling) then you become proud. It actually boosted our confidence in an emergency itself. When we qualify, we won’t shy away.’ (FG2, P1, female)

## Discussions

### Phase 1

The observers felt that by watching their peers participate in the simulation experience, they would also know what to do should such an emergency occur in a real-life situation. According to O’Regan et al. (2016), students value observer roles as it affords them an overview of details, and therefore observers are able to provide meaningful feedback during debriefing sessions. High levels of realism, during the high-fidelity simulation experience, forces students to engage actively, and according to Ness et al. (2010) and Neill and Wotton (2011) where the simulation scenario is authentic, students will act as if the situation is real.

Likewise, the *concrete experience*, as described by Kolb (2014), involves doing a task or having an experience. In this first phase of the experiential learning cycle, student midwives were able to ‘feel’ the experience by becoming actively involved in the situation. Previous studies, which used nursing students in complex high-fidelity simulations scenarios, also showed improvements on all learning outcomes, including critical thinking, confidence, competence and theory-practice integration (DeBourgh & Prion 2011; Jeffries & Jeffries 2012; Kaddoura et al. 2016).

It appears that by playing the role of a doctor in a team, there is an increased expectation from team members and this demands that you should know what to do in an emergency. Fry, Ketteridge and Marshall (2015) and Hill (2017) also saw similar results where students found the simulation experience to be fun and interactive as well as stressful and challenging. Role-playing in an obstetric emergency can be very challenging for students because of the complexity and urgency of the situation; however, when students are faced with an emergency, they may ‘give of their best’ and showcase a combination of skills to save the life of a patient. Allowing students an opportunity to play different roles in a simulation exercise can encourage them to understand how different people feel and respond in a real-life situation such as in the role of a doctor, a team leader, a student midwife or a birth companion. It also teaches students the importance of working quickly and harmoniously during an obstetric emergency to save the life of the patient.

### Phase 2

According to Neill and Wotton (2011), simulation debriefing is central to the simulation event as it guides students through a reflection of what just occurred. Similarly, in the phase of reflective observation, in the experiential learning cycle, the learner takes time out from the task or experience to review what was experienced (Wain 2017). In this study, the results of the focus groups showed that the scenario caught the full attention of the observers who were fully engaged throughout these discussions. Student midwives who observed the simulation scenario were able to evaluate the simulation exercise, the performance of the role-players and their own gaps and strengths. Reflection and feedback promote the integration of theory and practice through the development of critical thinking, and when students reflect on their experience, it enriches experiential learning (Butler, Veltre & Brady 2009; Lestander et al. 2016; McCormick 2014).

### Phase 3

The participants were able to identify areas that needed improvement and felt that they would know what to do if they experience a similar situation in reality. These feelings of self-confidence and self-worth develop when students come to the realisation that they can apply their knowledge and make clinical judgements (Mikasa, Cicero & Adamson 2013; Rourke, Schmidt & Garga 2010). According to Kolb (2014), the *abstract conceptualisation phase* occurs when students make their own theories or conclusions about an experience. They attempt to make sense of what occurred and interpret the events of the experience to promote understanding (Fry et al. 2015; Hill 2017). Similarly, the debriefing of this simulation scenarios encouraged student midwives to critically reflect on their experience of learning and make their own assumptions about their knowledge, skills and abilities. Comments related to both the positive and negative aspects, and showed that student midwives were aware of important principles in nursing care. Lapkins and Levett-Jones (2011) attest that simulation provides students with a chance to develop clinical judgement and affords the faculty an opportunity to observe the students’ clinical decision-making and practice in a confined, prescribed setting. Students who participate in simulation are forced to function at a higher cognitive level when they are faced with fast thinking and acting scenarios (Goodstone et al. 2013). According to Wain (2017), reflective practice increases self-awareness, self-identity and personal growth that lead to job satisfaction and professional fulfilment.

### Phase 4

It seems as if that prior to this session, participants felt less competent to be involved in procedures encountered in the clinical area. According to Kolb, the *active experimentation* phase involves the application of new theories and principles during decision-making and problem-solving activities. Wain (2017) describes that in this phase the learner applies what was learned into practice. Post-partum haemorrhage is a common obstetric emergency that requires a combination of these activities to manage a patient successfully and practising these skills helps student midwives to feel less anxious and more confident when faced with similar real-life situations. In this study, student midwives were fortunate to experience a similar scenario in real life and they felt confident to assist in the management of the patient. These results were mostly evident in the second focus group discussion, which revealed that student midwives are able to test or try out a combination of skills (practical, decision-making, problem-solving, critical thinking and clinical judgement) confidently in a real situation transferring the same principles of care used during the simulation experience.

High-fidelity human patient simulators used in complex scenarios offer student midwives the opportunity to manage real-life emergencies, reflect on their experience, evaluate how the experience has contributed to their learning outcomes and then incorporate the same principles or theories in a new situation. Through experiential learning and the use of high-fidelity simulation experiences, a link between classroom learning and the real working world becomes possible (Kolb 2014). High-fidelity human patient simulators used in a skills laboratory offer students an arena to practise their skills in certain procedures before actually conducting these procedures on real-life patients. This can be a good way to assist nurse educators to teach clinical skills which are difficult to teach in busy clinical placements or where clinical placement is limited (Berragan 2011).

Bergsteiner and Avery (2014), in their study, critically evaluated Kolb’s experiential learning cycle and highlighted the divergent and overlapping classifications of learning activities with other philosophers such as Svinicki and Dixon (1987), Butler (1986) and Fleming (2001). This resulted in the reconceptualisation of Kolb’s theory to a twin-cycle experiential learning model (TCELM) which accommodates both a concrete, active, primary learner and an abstract, passive, secondary learner. However, this new model was not used in a high-fidelity simulation experience previously.

### Summary

High-fidelity human patient simulators allow nurse educators an opportunity to integrate Kolb’s four-phase experiential learning to teach obstetric emergencies. The findings of this study are aligned to Kolb’s experiential learning cycle and described as follows:

- The concrete experience – HFHPS offers student midwives an opportunity to experience and manage real-life emergencies.
- Reflective observations – HFHPS promotes an opportunity to reflect on the experience.
- Abstract conceptualisation – HFHPS allows student midwives to think about the learning experience.
- Active experimentation – HFHPS allows student midwives to act/try out what was learned.

### Recommendations

High-fidelity simulation as a learning strategy has many possibilities for clinical nursing, and the impact of high-fidelity simulation on clinical competence should be explored in future studies.

Evaluating the outcomes of HFHPS used for teaching purposes and its effects on actual patient care in terms of quality is also essential.

### Limitations

The participants were known to the researcher, who was their teacher and clinical facilitator, and this may have influenced their responses. The study was limited to one setting.

## Conclusion

High-fidelity human patient simulators allow nurse educators a platform to promote experiential learning in a safe environment and decrease the challenges faced in clinical accompaniment. Although some criticism exists against Kolb’s experiential learning theory, it was well suited in this study to show how high-fidelity simulation in a PPH scenario promotes the four-phase cycle of experiential learning to role-players and student observers.
